# Multi-fractal detrended cross-correlation heatmaps for time series analysis

**DOI:** 10.1038/s41598-022-26207-w

**Published:** 2022-12-15

**Authors:** Paulo Roberto de Melo Barros Junior, Kianny Lopes Bunge, Vitor Hugo Serravalle Reis Rodrigues, Michell Thompson Ferreira Santiago, Euler Bentes dos Santos Marinho, Jose Luis Lima de Jesus Silva

**Affiliations:** 1 Petrobras, Petróleo Brasileiro S.A., Av. República do Chile, no 65 Centros, Rio de Janeiro, 20031-912 Brazil; 2 UniFAMEC, Avenida Leste, Ponto Certo, Camaçari, BA 42801-170 Brazil; 3 Geological Survey of Brazil - CPRM, Avenida Ulysses Guimarães, 2862 Centro Administrativo da Bahia, Salvador, BA 1649-026 Brazil; 4 Polytechnic School of Engineering and Research Center in Geophysics and Geosciences, Federal University of Bahia, Rua Barão de Jeremoabo, Ondina, Salvador, BA 40210-630 Brazil; 5 Division of Artificial Intelligence and Integrated Computer Systems, Department of Computer and Information Science, Linköping University, Linköping, 581 83 Sweden

**Keywords:** Engineering, Mathematics and computing

## Abstract

Complex systems in biology, climatology, medicine, and economy hold emergent properties such as non-linearity, adaptation, and self-organization. These emergent attributes can derive from large-scale relationships, connections, and interactive behavior despite not being apparent from their isolated components. It is possible to better comprehend complex systems by analyzing cross-correlations between time series. However, the accumulation of non-linear processes induces multiscale structures, therefore, a spectrum of power-law exponents (the fractal dimension) and distinct cyclical patterns. We propose the Multifractal detrended cross-correlation heatmaps (MF-DCCHM) based on the DCCA cross-correlation coefficients with sliding boxes, a systematic approach capable of mapping the relationships between fluctuations of signals on different scales and regimes. The MF-DCCHM uses the integrated series of magnitudes, sliding boxes with sizes of up to 5% of the entire series, and an average of DCCA coefficients on top of the heatmaps for the local analysis. The heatmaps have shown the same cyclical frequencies from the spectral analysis across different multifractal regimes. Our dataset is composed of sales and inventory from the Brazilian automotive sector and macroeconomic descriptors, namely the Gross Domestic Product (GDP) per capita, Nominal Exchange Rate (NER), and the Nominal Interest Rate (NIR) from the Central Bank of Brazil. Our results indicate cross-correlated patterns that can be directly compared with the power-law spectra for multiple regimes. We have also identified cyclical patterns of high intensities that coincide with the Brazilian presidential elections. The MF-DCCHM uncovers non-explicit cyclic patterns, quantifies the relations of two non-stationary signals (noise effect removed), and has outstanding potential for mapping cross-regime patterns in multiple domains.

## Introduction

Economic scenarios are complex^1–3^, and their mechanisms are challenging^4–7^. According to Hidalgo^8^, the study of economic complexity has accelerated in recent decades not only focused on traditional approaches to aggregate outputs such as gross domestic product (GDP), capital, labor, and knowledge. Complexity metrics can provide robust descriptors of diversification and indications of potential development for diverse economies and markets^9^. The study of economic complexity has also grown with the revival of industrial policy^10,11^, endogenous growth theory^12–17^, and quantitative studies of complex economic structures in numerous countries^18–24^. According to Arthur^25^, the neoclassical^26^ theory assumes rational agents with well-defined problems arriving at optimal behavior in equilibrium with the overall outcome, which can be unrealistic for markets^27^ since it does not take into account possible anomalies, market phenomena, bubbles, crashes, and random periods of volatility. However, complexity economics assumes that agents differ from each other, explore, react and change actions in response to mutual outcomes. Consequently, patterns and emergent phenomena can be displayed out of equilibrium. These systems can generally exhibit complexity^28–35^, adaptive and non-linear dynamic behavior^36,37^. Therefore, their overall properties cannot be easily inferred from isolated components^38,39^. Complex systems can also present emergent properties^40–42^ from large-scale exchanges, dependencies, and connections that only emerge when the system is all-together. The concept of spontaneous order and self-organization economy dates back to the founder of the Austrian School, Carl Menger, in his work regarding spontaneous emergence of money transactions in primitive economies^43^. Hayek^44^ extended the concept to self-organization phenomenon with emergence and complexity^45,46^, followed by the support of Koppl^47,48^, Rosser^49^ and Lewis^50^.

Attributes such as market competitiveness^51^, continuous variations of exchange currency rates^52^, Gross domestic product^53^ (GDP), interest rates (IR), disposable incomes^54^ (DI), and global consumer demands can significantly impact one’s organization. These dynamic aspects always lead to internal and external implications since actors should consider cost-effective strategies to mitigate financial risks^55^, maximize profits, and encourage growth^56^. However, elements of individual actions can generally propagate through economic networks with critical topologies for the stability of economies^57^, self-organization^58^, and risk propagation. These economic networks can cause a cascade of events^59^ with long-tailed distributions characterized by power laws. These power laws are expected in economies, and markets due to volatilities and fluctuations in prices^60–62^. Therefore, comprehending the relationship between internal and external signals and fluctuations can be critical for appropriate planning since disruptive economic scenarios can lead to unstable institutions.

The time series analysis of pairs of signals and fluctuations can assess possible data-driven persistences (the tendency of a system to remain in the same trending state), anti-persistences (the tendency of a system to remain in opposing trending states), general trends and its cyclical patterns. Institutions can use these tools to improve efficiency and productivity throughout their supply chain by planning and executing short, medium, and long-term strategies to avoid disruptions. For instance, measuring the cyclical fluctuations of sales and inventory could help prevent the underproduction of a particular product during a period. However, the common bottleneck is how to effectively track seasonal (up to 1 year) trends and cyclical patterns from time-evolving fluctuations. Previous studies have shown the potential of cross-correlation analysis for decision-making across multiple fields. The detrended cross-correlation analysis (DCCA) has been used to investigate possible power laws over prices and volume changes in the stock market^63,64^. The methods section clarifies that the DCCA generalizes the standard covariance to consider the long-range memories of two non-stationary signals. Besides, the DCCA has also been used in climatology to track the influence of seasonal patterns^65,66^.

The signals can have scale invariance when an internal structure repeats on subintervals of the same signal. Therefore conventional methods such as the moving averages cannot capture invariant signatures. For instance, given a time-evolving signal $X(ct)=c^{\alpha } X(t)$, we can estimate the power law exponent $\alpha$ using fractal analyses to define the kind of scale-invariant structure it possesses. These scale-invariant structures are also widely found in biomedical signal processing. They can support the prognostic and diagnostic of patients since any alteration of exponents could reflect the adaptability and success of a treatment to improve pathological conditions and health^67,68^. A single power law exponent assumes that the scale invariance is independent of time and space. However, spatial and temporal variations indicate a multifractal structure, which means a spectrum with multiple power law exponents. These scaling factors (exponents) can provide information about hidden cyclical regimes ranging from months to years. Multifractals are widely used in Finance to investigate financial time series across different markets and assets^69,70^. The multifractal detrended fluctuation analysis (MF-DFA)^71^ has been used to investigate the hedging effectiveness of Chinese treasury bonds and interest rate risk^72^. Furthermore, the multifractal detrended cross-correlation analysis (MF-DCCA) can analyze self-similarities levels between Shanghai and Hong Kong Stock markets^73^. There have been multiple advances in the domain with new tools such as the DCCA-$\textit{l}(n)$^74^, random matrix-based DCCA for time-delay cross-correlation^75^, and combinations with Support Vector Machines (SVM) to forecast financial returns^76,77^. Additionally, Graphs using DCCA (cross-correlation between nodes) have been used to model financial networks, analyze stock exchanges, market hubs, cluster community centrality, and connection between networks^78^. Therefore, an extension of the MF-DCCA capable of mapping cross-correlations for multiple regimes in a single heatmap can be highly advantageous for decision-making across multiple domains.

We propose the multifractal detrended cross-correlation heatmaps (MF-DCCHM) based on the DCCA cross-correlation coefficients with sliding boxes accounting for 5% of the entire series. It is crucial to use the integrated series of magnitudes and averages of DCCA coefficients on top of the heatmaps for the local analysis. This systematic approach can map the overall relationships between fluctuations of signals on different time scales and multifractal regimes (different fractal dimensions). This method uncovers non-explicit cyclic patterns, quantifies the relations of two non-stationary signals, and can stand out as a potential approach for applications in multiple domains. For the present work, we have used time series of inventory and sales extracted from the National Federation of the Distribution of Motor Vehicles (FENABRAVE) database registered from 1995 to 2020 extracted from the Central Bank of Brazil^79^. Forecasting sales and stocks in the automobile sector are of utmost importance to decision-makers engaged in resource allocation on the supply side. The Brazilian Automotive sector held a share of 22% of industrial production and 4% of the total GDP in 2018. The sector is also responsible for 1.6 million employees and pays around 40 billion dollars yearly in taxes. The Brazilian Automotive Industry was also classified in the same group as the USA and South Korea regarding market structure^80^. The latter elucidates the size of the economic impact and the importance of accurate models and systematic approaches to forecasting sales in an economic context. Time series analysis and data mining algorithms^81–83^ along with neural networks, fuzzy analysis, and multiple linear regressions^84^ have been used to forecast sales. However, only linear regression models^85^ were used to find elasticities for sales in the Brazilian Automotive sector. Therefore, we have explored the analysis of the cross-correlation between sales and inventory from the Brazilian Automotive sector and growth descriptors such as the gross domestic product (GDP) per capita, the nominal exchange rate (NER), and the nominal interest rate (NIR) equivalent to the Special Settlement and Custody System (Selic). The following approaches were used for the analysis: (i) the detrended fluctuation analysis (DFA) for auto-correlation and detrended cross-correlation analysis (DCCA) to estimate possible trends and concurrent events that might affect decision-making processes, (ii) the cross-correlation coefficients (CCC) to verify the level of correlation for different periods, (iii) Discrete Fourier analysis to identify, distinguish, and characterize the various cycles, and (iv) MF-DCCHM to evaluate cyclic patterns from a pair of time-evolving signals. Our global analysis has shown anti-correlated patterns from fluctuations in sales and inventory, which can help identify scenarios for the automotive sector. We have also assessed cyclical patterns on time series using the MF-DCCHM method. This technique indicates positively correlated patterns from our dataset that can be directly compared with the amplitude and power-law spectra. We also show critical cyclical patterns and regimes of high intensities that coincide with the Brazilian presidential elections. This work presents the advantages of employing the MF-DCCHM method for capturing cyclical trends to guide decision-making processes. The existing methods DFA and DCCA^63,86^ only present a global estimate based on scaling exponents to the level of autocorrelation and cross-correlation between the series. It is only possible to access information on a series of fluctuations for its entire length since there is no sliding box, and the cross-correlation coefficients are only functions of window size^65^. The novelty of our work relies on local analysis. The local analysis (MF-DCCHM) can uncover cyclical patterns, anomalies, persistences, and anti-persistences of signals for small intervals of the series in different multifractal regimes (exponent factor). This systematic approach is more effective using a series of magnitudes^87^. The analysis is carried out by choosing a subseries (short interval) of the signal (sliding box with size up to 5%), which is swept (similar to a Moving Average) for the full extension of the series and fluctuations. Therefore, the mapping considers all scales/periods of the fluctuations. Hence, unlike existing techniques, which depend only on scale variation, our systematic approach computes the cross-correlation coefficient considering scale variation and temporal/spatial variation. The use of DCCA cross-correlations with sliding boxes to build heatmaps was first introduced by Marinho et al.^88^. Recent works^89,90^ use a similar approach for mapping the DCCA cross-correlation coefficients considering integrated series and sliding boxes with a size of 50% of the entire series. However, our work takes advantage of an integrated series of magnitudes with non-linear properties that amplify the signals. This step is crucial for uncovering anomalies and cyclical patterns across multifractal regimes in the maps. Furthermore, the maps will not uncover patterns from multiple multifractal regimes if the size of the sliding box is significantly large, for instance, 50% of the entire series, as highlighted by the references^89,90^. Our contribution also relies on the fact that depending on the sampling, the sliding box must have a size of up to 5% of the entire series. The spectral analysis confirms this fact since we found the same cyclical frequencies across different multifractal regimes in our heatmaps. We cannot find the same results by considering only the integrated series with a sliding box accounting for 50% of the entire series. Furthermore, it is also possible to use the approach with a configurational space that is not dependent on time, which is highly beneficial and efficient for pattern recognition in numerous domains. The methods section details on stochastic methods and spectral analysis underlying our fluctuation analysis.

## Results

### Global analysis to estimate multifractal exponents

In the pre-processing phase, each time series correspond to signals composed of short to long-range stationary intervals. Therefore, the first step was statistically mapping non-stationary time series into stationary ones. The procedure is to establish the successive differences of the original series until its convergence to stationarity, as shown in Fig. S1 (Supporting Information). Additionally, we have computed the cumulative sum of the series of increments by considering consecutive differences of discrete points to reach the fully integrated series. Figure S2a,b (Supporting Information) shows a time series of sales in units (Brazilian Automotive sector) and their successive differences, respectively. Figure S2c (Supporting Information) shows a step series derived from the magnitude. The integrated-time series $y_k^M$ defined by $M = | x_i |$^91,92^ was computed from Eq. (5), as shown in Fig. S2d (Supporting Information). We compute the decomposition of $y_k$ into a series of magnitudes as a useful strategy to characterize fluctuations and patterns obtained from the original series after computing $F_X$ from Eq. (4). Using the series of magnitudes and sliding boxes, we have performed a local analysis by computing the coefficients $DFA_1$ and $DCCA_1$. As shown in Fig. S3a (Supporting Information), we have chosen one box *s* (a subset of the series) of a given size $N'$ to compute $\sigma _{DCCA_1}$. The box *s* is divided into $M_v$ windows, each with size *v*, as shown in Fig. S3b (Supporting Information). We have also obtained the autocorrelation and cross-correlations of $y_k$, and $y_k'$ in the interval $N'$, and $F_X$ to solve Eq. (12). After computing these coefficients, the sliding boxes move forward while keeping the same number of windows ($M_v$) to extract the new coefficients $\sigma _{DCCA_1}$^87,88^. We repeat this procedure multiple times for sampling the time series of size *N* with the same box *s*. Finally, the box of size $N'$ returns to the starting point for different windows of size *v*. The goal is to map the fluctuations with different windows of size *v* to obtain the coefficients $\sigma _{DCCA_1}$ as a function of the scaling factor *v* and time using the correlation heating maps. This mapping is crucial because it sweeps across multiple regimes and unveils invariant structures across different multifractal exponents. We will now refer to the new multifractal detrended cross-correlation heatmaps as the MF-DCCHM method.

**Figure 1:**
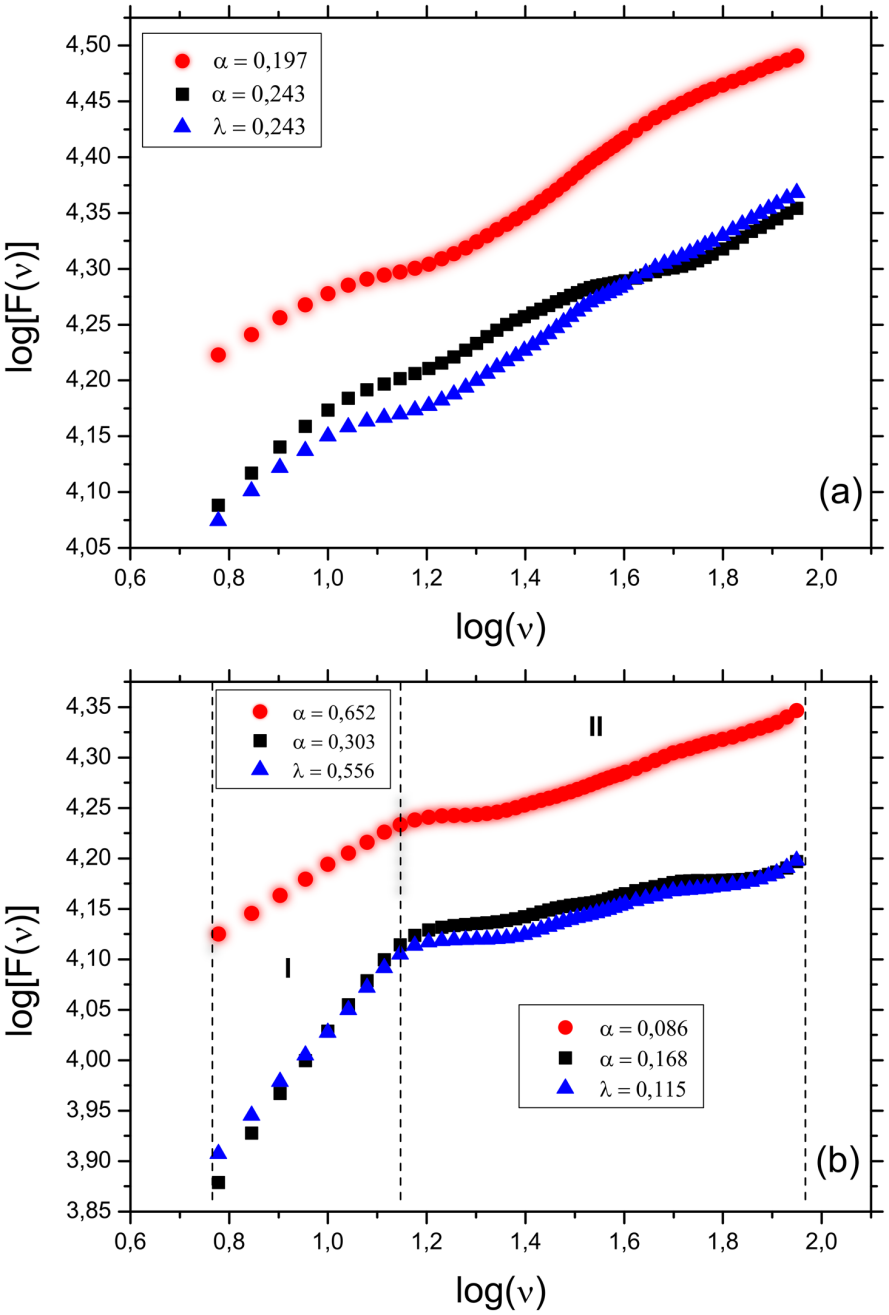
Fluctuation analysis using the following references: (**a**) average and (**b**) trend. The sales, inventory, and sales & inventory are represented by circles (red), squares (black), and triangles (blue), respectively. Regions I and II indicate different q-Multi-fractal regimes, where q = 2.

Figure 1a and Table 1 shows a linear trend, and both exponents from the autocorrelation and cross-correlation are lower than 0.5. These characteristics indicate that fluctuations around the average tend to reverse in the future. Therefore, in the time interval from 1995 to 2010, the exponents reveal that Brazilian car sales and inventory fluctuations will reverse their growth direction. We have observed a similar scenario from the cross-correlation exponent, where the fluctuations of sales and inventory follow different directions. From 1995 to 2016, we can observe the highest positive fluctuations from the first 15 years, followed by a downward trend as the sales dropped drastically. Figure 1b and the exponents in Table 2 show a q-Multifractal (q = 2) with two regimes and a crossover pattern on the threshold $\log {(v)}=1.15$, where $v\approx 14$ months. Region I shows that the exponent for the autocorrelation of sales and inventory are anti-persistent and persistent, respectively. However, Region II shows that all exponents have lower values than 0.5, indicating that future fluctuations will reverse direction. Additionally, Fig. 5b shows that for periods greater than $v = 14$ months, the pattern of fluctuation around the linear trend is very similar to the fluctuations around the mean from Fig. 1a.

**Table 1:** $DFA_0$ and $DCCA_0$ exponents for the sales and inventory time series.

Sales ($\alpha$)	Inventory ($\alpha$)	Sales and inventory($\lambda$)
0.243 ± 0.004	0.197 ± 0.004	0.243 ± 0.003

**Table 2:** q-Multifractal $DFA_1$ and $DCCA_1$ with q = 2 exponents for the sales and inventory time series.

Region	Sales ($\alpha$)	Inventory ($\alpha$)	Sales and inventory($\lambda$)
I	0.303 ± 0.003	0.652 ± 0.008	0.556 ± 0.005
II	0.168 ± 0.001	0.086 ± 0.003	0.115 ± 0.002

### Local analysis to estimate the coefficients DFA$_1$ and DCCA$_1$

**Figure 2:**
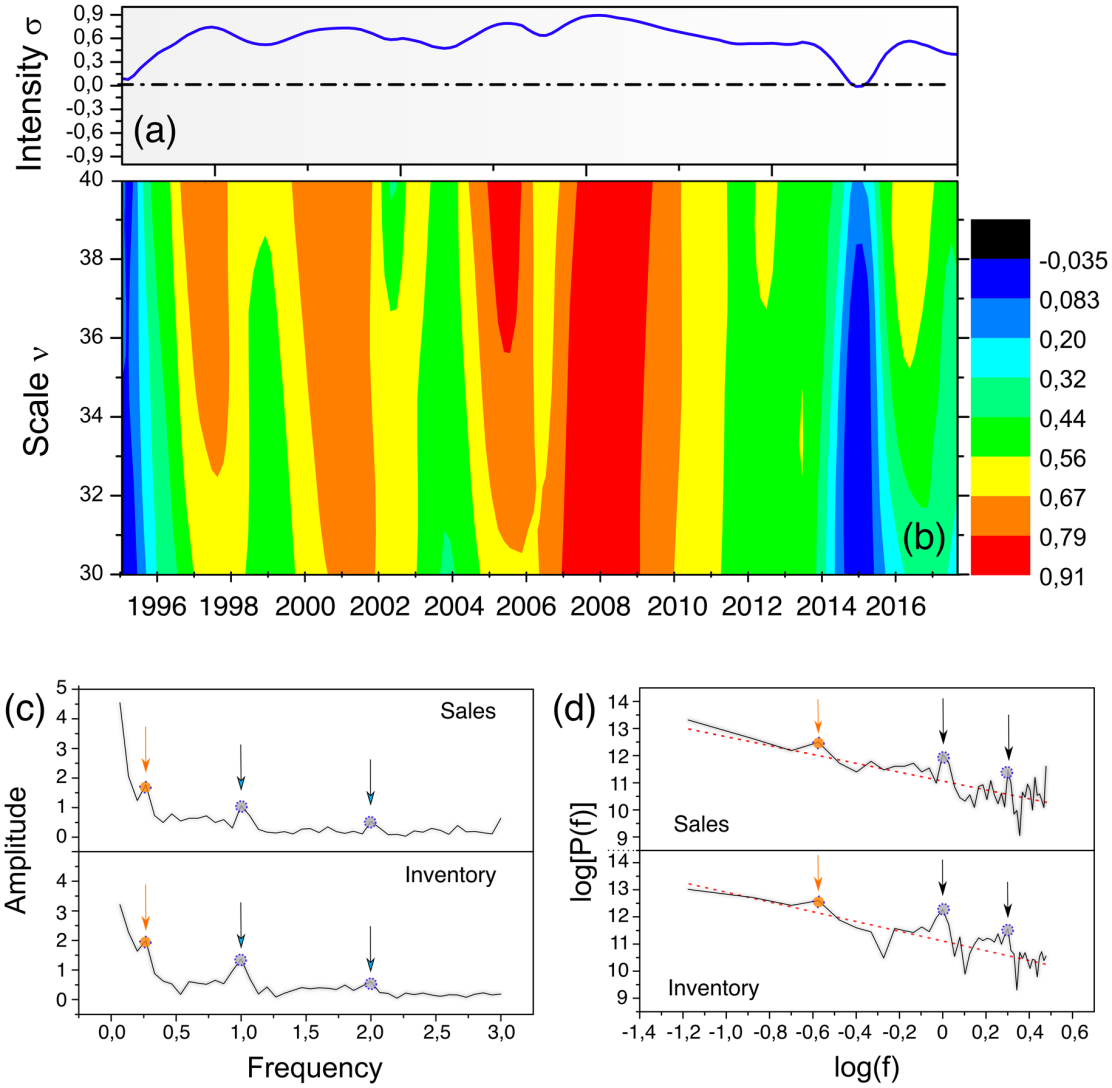
MF-DCCHM diagram of cross-correlations. (**a**) Average for each period. (**b**) MF-DCCHM heatmap of cross-correlations considering multiple sliding boxes *v* distributed over the years. (**c**) The amplitude spectra and (**d**) power versus annual frequency for the inventory and sales.

Figure 2a shows the MF-DCCHM sampled over multiple windows of size *v* and times. This heatmap has distinct colors assigned according to the magnitude of the detrended cross-correlation coefficients, namely $\sigma$. On top of each map, we have also plotted an average of $\sigma$ (y-axis) for each time. Figure 2a show several multi-fractal regimes captured by distinct coefficients within the interval $0<\sigma <1$ as a function of the window *v*. The fluctuations of the two series have shown a positive cross-correlation with periodic high-intensity patterns. We can distinguish intermittent anomaly levels with greater intensities associated with major concurrent events, particularly for $\sigma > 0.6$. We have also found a cycle with an average of approximately three to four years for $\sigma > 0.85$. During this period, the Brazilian automotive sector retained high incentives, subsidies, investments, and a compelling sales season throughout the automotive industry. Additionally, the intensity of positive cross-correlations increases across multiple governments during the same cycle, with a maximum threshold between 2003 and 2004. Figure 2b confirms our previous assumptions of a three to 4-year cyclic pattern (Presidential Elections). This periodicity shows that the fluctuations for the two series have high degrees of similarity.

Our results suggest that the automotive sector was optimized with neither underproduction nor overproduction. Figure 2b confirms our previous three- to four-year cycle assumptions. To conclude, visualizing a heating map sweeping $\sigma$ for multiple window sizes for different years can unveil hidden patterns, such as the rate and variability between two-time series. We can also detect soft-to-strong trends and possible impacts on one’s companies’ production lines, as optimized sales and inventories can lead to higher revenues. Therefore, this approach can be of interest to strategic planning and decision-making.

### Analysis of the amplitude and power spectra

The time series were sampled with the annual interval to obtain the amplitude and spectra of frequency from 1995 to 2010 from the following equation:

1 $$\begin{aligned} \Delta f = \frac{1}{N\Delta t} = \frac{1}{180 \times \frac{1}{12}} \approx 0.067 ~cycles/year. \end{aligned}$$

Figure 2c,d shows the amplitude and power spectra for the sales and inventory time series. In Fig. 2c we observe two outstanding seasonal peaks with annual periodicity (f = 1 cycle/year) and semi-annual periodicity (f = 2 cycles/year). The latter represents an annual cycle of higher demands for vehicles and periods with lower-order intensity, respectively. The frequency peak in the proximity of f = 0.25 cycles/year corresponds to approximately 4 years. Additionally, Fig. 2d presents the power spectra where we can identify the same peaks from Fig. 2c. The coefficients *b* for sales ($1.7 \pm 0.2$) and inventory ($1.6 \pm 0.1$) are in the range $-1<b <3$, which indicates a strong cross-correlation. For instance, the peak over the frequency f = 0.25 cycles/year coincides with the MF-DCCHM shown in Fig. 2a for regimes where $\sigma > 0.85$. Suppose $b = 0$, the series of increments would be equivalent to random series. However, our results suggest important events with distinct periodic demands. This technique can also detect seasonal anomalies and larger cycles to support strategic planning and higher decision-making confidence at multiple levels.

### MF-DCCHM analysis

**Figure 3:**
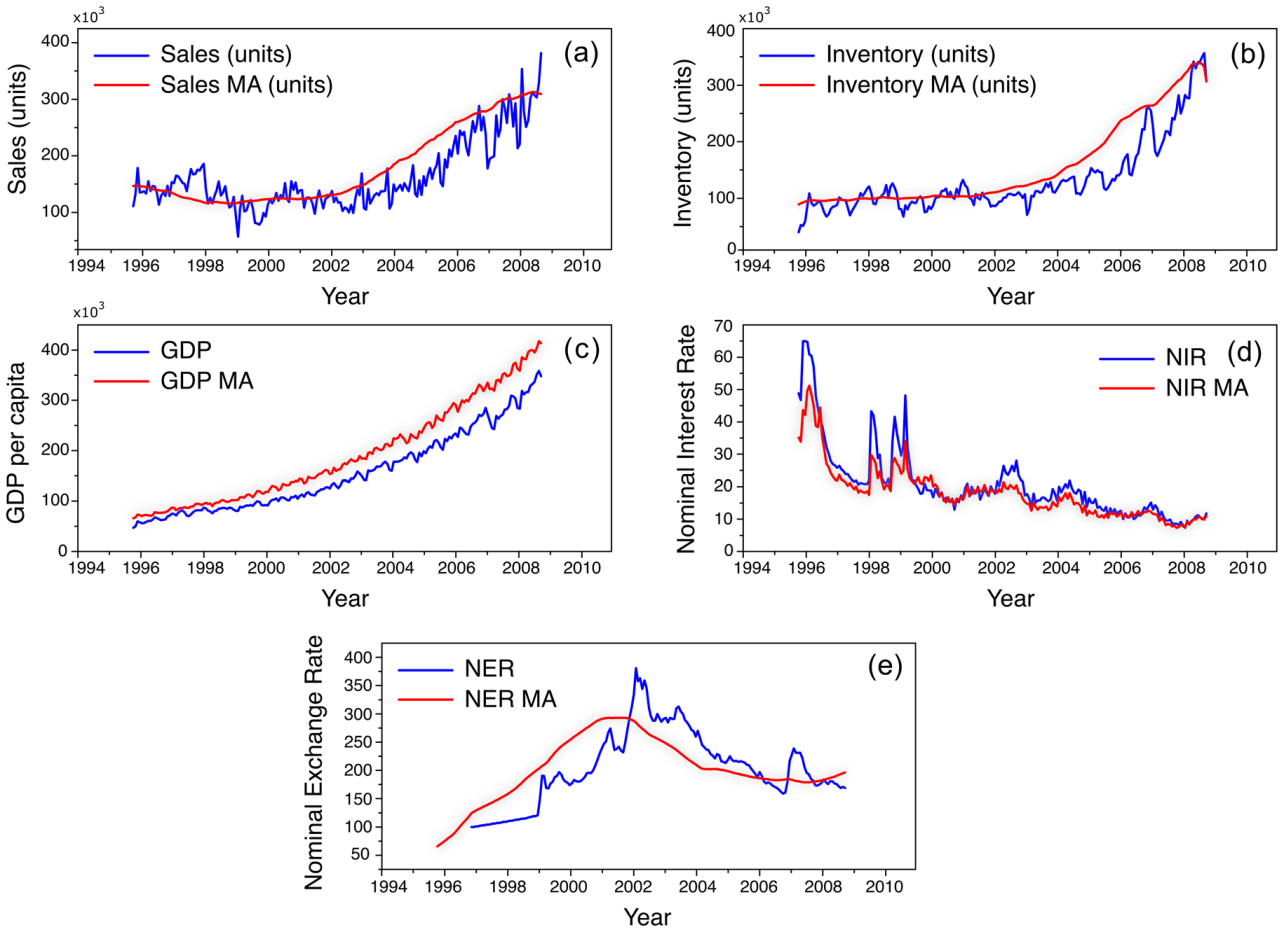
Time series (blue) and moving average (red) of (**a**) Sales (Units) and (**b**) Inventory (units) from the Brazilian Automotive Sector, and Brazilian (**c**) GPD per capita, (**d**) Nominal Interest Rate, and (**e**) Nominal Exchange Rate.

We have also investigated if growth descriptors would show cyclical patterns when cross-correlated with the fluctuations in sales of the Brazilian automotive sector. Figure 3 shows the value and its respective Moving Average (M.A.) for national (a) sales and (b) inventory of automotive vehicles in units, (c) Gross Domestic Product per capita (GDP per capita), (d) Nominal Interest Rate (NIR), and (e) Nominal Exchange Rate (NER) obtained from the Brazilian Central Bank^79^. We have used the Brazilian GDP per capita, a metric of economic activity and output by its total population, and consider the monetary worth of goods and services per month, an essential variable indicative of a country’s living standard. The analysis of M.A. shows expected growth for the sales and inventory when the GDP per capita increases and the NIR reduces.

After the introduction of the Real in 1994, Brazil had a substantial influx of international capital leading to a gain in the value of the Real against the U.S. dollar leading to reasonable economic stability. From 1996 to 1998, Fig. 3 shows that the moving average of NIR decreased while NER smoothly increased due to the reduction of the tight control of the exchange rates by the Central Bank of Brazil^79^. Furthermore, from 1998 to 2002, Brazil faced high inflation and the prospect of political risk due to upcoming elections led to an outflow of capital, high volatility of NIR, and a peak of NER around 2002. These aspects are essential instruments for decision-making management. However, the moving average is insufficient to capture trends regarding the cyclic patterns and fluctuations between macroeconomic time series. Therefore, we have developed the MF-DCCHM method and employed it as an alternative to understanding the variability, trends, persistences and anti-persistences, and dependencies among these macroeconomic indicators and their possible effects on the Brazilian automotive sector.

**Figure 4:**
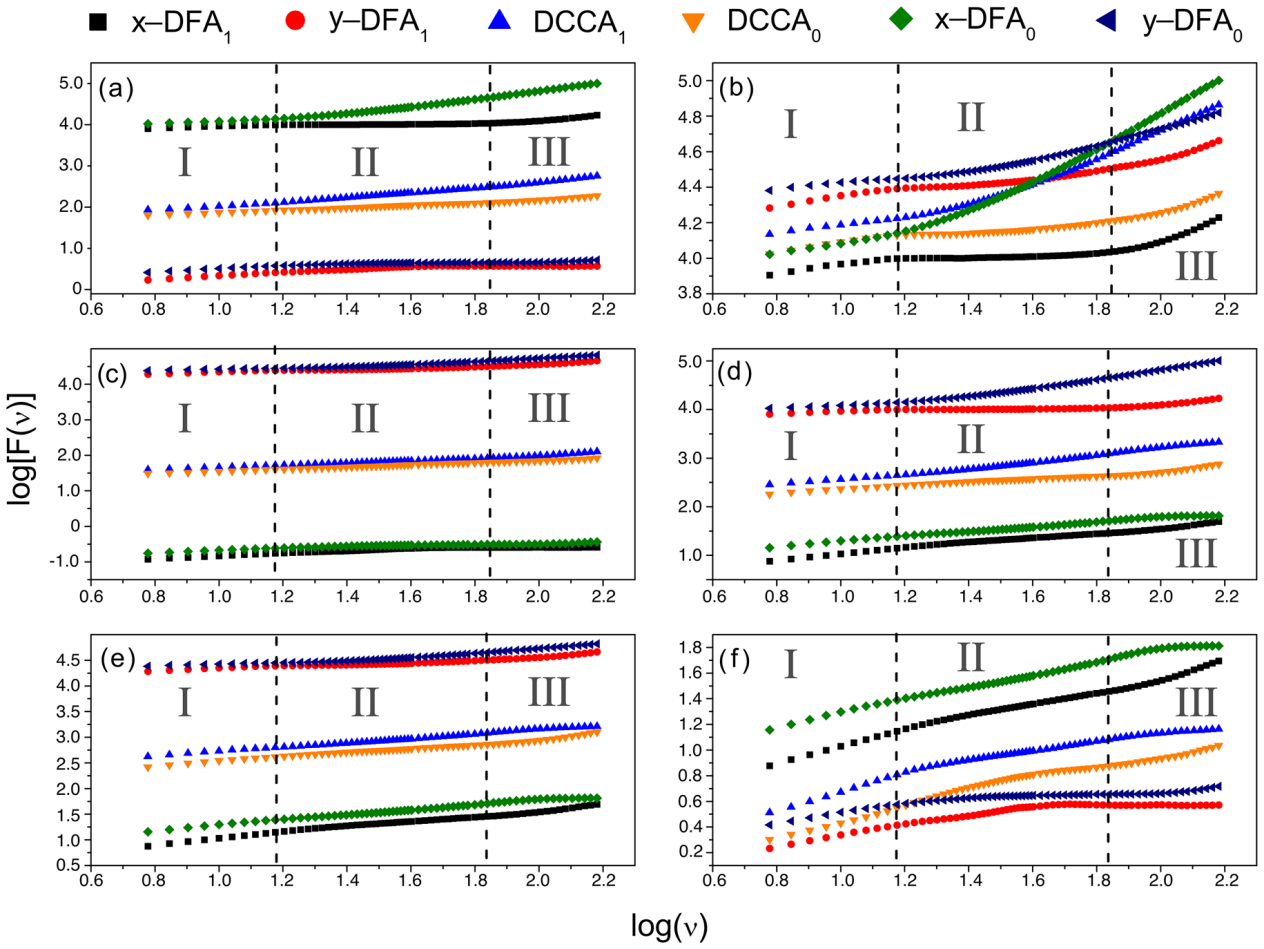
Fluctuation analysis with three multifractal regimes for (**a**) NIR and GDP, (**b**) Sales and GDP, (**c**) Sales and NIR, (**d**) NER and GDP, (**e**) Sales and NER, and (**f**) NER and NIR.

Figure 4 shows the fluctuation analysis for each pair of time series as a function of the period $\nu$. We can observe a multifractal regime with a crossover of three regions with different exponents (scaling factors) characteristics of soft-to-strong variability rates over time. The breaking of the first regime from Region I to Region II, occurs in approximately 15 months and from Region II to Region III in nearly 67 months. These regimes can be crucial to forecast the persistence of autocorrelation and cross-correlation trends for the pair of time series. The exponents derived from the global analysis for each pair of time series are shown in Tables 3, 4, 5, 6, 7 and 8. We have found positive persistence for the autocorrelation (DFA) of NER in Region I, GDP for Region II, and NER and GDP for Region III. This result is crucial for planning and an efficient decision-making process, given that persistence of NER and GPD for seasonal regimes can directly impact sales. The DCCA exponent increases from Region I (anti-persistent) to Region III (persistent), except for the DCCA exponent from NER and NIR (Table 8), which decreases from Region I (persistent) to Region III (anti-persistent). The latter means a direct relationship exists between the period value $\nu$ and the detrended cross-correlation exponent.

**Table 3:** Cross-correlation exponents considering the GDP per capita and nominal exchange rate (NER).

Method/time-series	Region I	Region II	Region III
DFA$_1$/NER	0.674 ± 0.003	0.444 ± 0.004	0.71 ± 0.02
DFA$_1$/GDP	0.23 ± 0.01	0.049 ± 0.003	0.58 ± 0.02
DFA$_0$/NER	0.59 ± 0.01	0.473 ± 0.005	0.28 ± 0.03
DFA$_0$/GDP	0.292 ± 0.007	0.80 ± 0.01	0.999 ± 0.003
DCCA$_1$	0.43 ± 0.01	0.299 ± 0.004	0.74 ± 0.03
DCCA$_0$	0.481 ± 0.005	0.680 ± 0.008	0.68 ± 0.01

**Table 4:** Cross-correlation exponents considering the sales in units and GDP per capita.

Method/time-series	Region I	Region II	Region III
DFA$_1$/GDP	0.23 ± 0.01	0.049 ± 0.003	0.58 ± 0.02
DFA$_1$/sales	0.300 ± 0.007	0.154 ± 0.005	0.49 ± 0.02
DFA$_0$/GDP	0.292 ± 0.007	0.80 ± 0.01	0.999 ± 0.003
DFA$_0$/sales	0.183 ± 0.009	0.308 ± 0.006	0.484 ± 0.004
DCCA$_1$	0.292 ± 0.008	0.111 ± 0.004	0.49 ± 0.02
DCCA$_0$	0.223 ± 0.009	0.556 ± 0.009	0.798 ± 0.002

**Table 5:** Cross-correlation exponents considering the sales in units and nominal exchange rate (NER).

Method/time-series	Region I	Region II	Region III
DFA$_1$/NER	0.674 ± 0.003	0.444 ± 0.004	0.71 ± 0.02
DFA$_1$/sales	0.300 ± 0.007	0.154 ± 0.005	0.49 ± 0.02
DFA$_0$/NER	0.59 ± 0.01	0.473 ± 0.005	0.28 ± 0.03
DFA$_0$/sales	0.183 ± 0.009	0.308 ± 0.006	0.484 ± 0.004
DCCA$_1$	0.49 ± 0.01	0.358 ± 0.002	0.73 ± 0.03
DCCA$_0$	0.44 ± 0.01	0.425 ± 0.004	0.36 ± 0.01

**Table 6:** Cross-correlation exponents considering the sales in units and nominal interest rate (NIR).

Method/time-series	Region I	Region II	Region III
DFA$_1$/NIR	0.418 ± 0.003	0.27 ± 0.01	0.021 ± 0.004
DFA$_1$/sales	0.300 ± 0.007	0.154 ± 0.005	0.49 ± 0.02
DFA$_0$/NIR	0.368 ± 0.005	0.139 ± 0.006	0.21 ± 0.02
DFA$_0$/sales	0.183 ± 0.009	0.308 ± 0.006	0.484 ± 0.004
DCCA$_1$	0.303 ± 0.006	0.318 ± 0.003	0.33 ± 0.01
DCCA$_0$	0.302 ± 0.001	0.304 ± 0.001	0.53 ± 0.01

**Table 7:** Cross-correlation exponents considering the GDP per capita and nominal interest rates (NIR).

Method/time-series	Region I	Region II	Region III
DFA$_1$/GDP	0.23 ± 0.01	0.049 ± 0.003	0.58 ± 0.02
DFA$_1$/NIR	0.418 ± 0.003	0.27 ± 0.01	0.021 ± 0.004
DFA$_0$/GDP	0.292 ± 0.007	0.80 ± 0.01	0.999 ± 0.003
DFA$_0$/NIR	0.368 ± 0.005	0.139 ± 0.006	0.21 ± 0.02
DCCA$_1$	0.296 ± 0.004	0.298 ± 0.004	0.50 ± 0.01
DCCA$_0$	0.432 ± 0.008	0.5735 ± 0.0004	0.81 ± 0.01

**Table 8:** Cross-correlation exponents considering the nominal exchange rate (NER) and nominal interest rate (NIR).

Method/time-series	Region I	Region II	Region III
DFA$_1$/NER	0.674 ± 0.003	0.444 ± 0.004	0.71 ± 0.02
DFA$_1$/NIR	0.418 ± 0.003	0.27 ± 0.01	0.021 ± 0.004
DFA$_0$/NER	0.59 ± 0.01	0.473 ± 0.005	0.28 ± 0.03
DFA$_0$/NIR	0.368 ± 0.005	0.139 ± 0.006	0.21 ± 0.02
DCCA$_1$	0.61 ± 0.01	0.47 ± 0.02	0.46 ± 0.01
DCCA$_0$	0.754 ± 0.007	0.377 ± 0.004	0.218 ± 0.009

**Figure 5:**
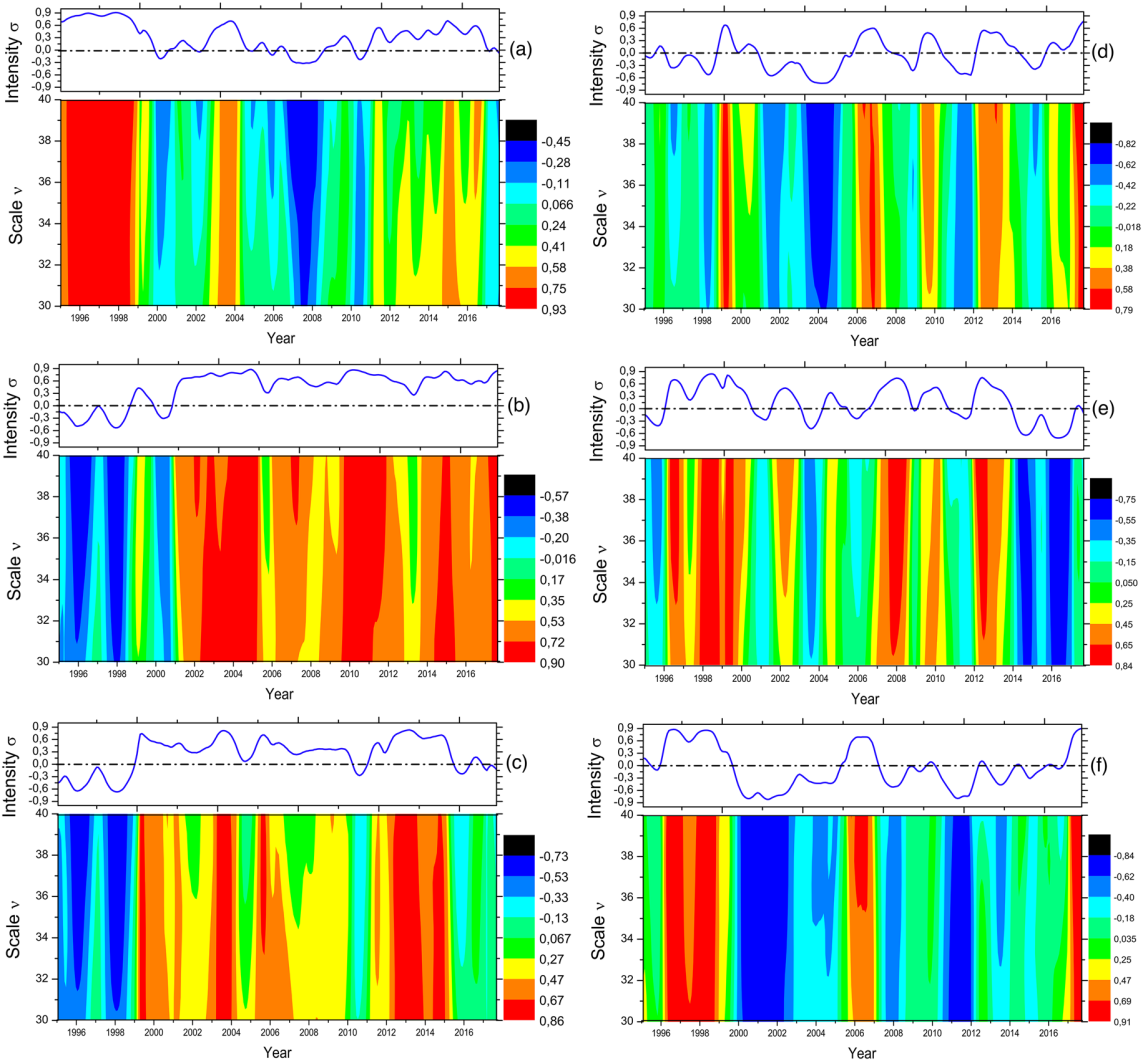
Multifractal detrended cross-correlation heatmaps of (**a**) NIR and Sales, (**b**) GDP and Sales, (**c**) NIR and GDP, (**d**) NER and GDP, (**e**) NER and NIR and (**f**) NER and Sales.

Figure 5 shows a local analysis of the raw time series’s fluctuations with the MF-DCCHM for six different time series mentioned at the beginning of this section. We have observed that all the maps have shown coefficients with intensities oscillating between − 1 and + 1, besides offering an averaged temporal periodicity that varies according to the adopted scale. Figure 5a shows two possible regimes where credit conditions can be relevant to sales with positive persistence between Sales and NIR from 2008 and periods where it can be irrelevant because the average intensity of $\sigma$ is closer to 0. We have confirmed the reliability of our approach since the GDP per capita, and Sales in the Brazilian automotive sector, as well as the GPD and NIR, have strong persistence, as shown in Fig. 5b,c. The average intensity of $\sigma$ on top of Fig. 5d,e shows cyclical patterns of 3 years with stable oscillations and persistence peaks around 2007, 2010, and 2013. Figure 5f shows an overall anti-persistence pattern compatible with the argument that unfavorable fluctuations in the NER tend to decrease the prices and possibly increase sales of automotive vehicles in Brazil.

## Discussion

We have developed a systematic methodology, namely the Multifractal detrended cross-correlation heatmaps (MF-DCCHM), to expose non-explicit patterns from time-series fluctuations with multiple regimes. Our study case explores cross-correlations between the sales and inventory derived from the Brazilian Automotive sector and growth descriptors such as the gross domestic product (GDP) per capita, the nominal exchange rate (NER), and the nominal interest rate (NIR) equivalent to the Special Settlement and Custody System (Selic). In the first part of this work, we obtained cross-correlation exponents using a global analysis to estimate the most probable scenarios in the Brazilian automotive sector after 2010. For the local analysis, it was possible to establish periodic intervals where the statistical variables sale and inventory have a substantial positive (window sizes $0<v<1$) cross-correlation. This approach has shown a cyclical pattern of high intensity with approximately three to four years, which coincides with periods near the presidential elections. We have also found cyclical frequencies over several periods through the application of Fourier analysis, where three primary seasonal cycles were identified: (i) semester, (ii) annual, and (iii) 4 years. We have shown that the fluctuations from later periods of 1995 to 2010 provide short to long-term predictability in demand over production lines, which stimulated an adequate performance during the highest and lowest sales season.

The MF-DCCHM detect non-linearities between macroeconomic time series and their effects on sales. The MF-DCCHM was able to bring insights into the persistence between the fluctuations. The trend in the correlation between GDP and NIR has a turning point around the year 2000 from a negative to positive persistence in the entire interval of window sizes. The positive persistence coincides with the high stability of the Brazilian economy. We observed a degrading effect around 2008 connected to the global financial crisis. For GDP and NER, we observe negative and positive persistence cycles, which can be interpreted as the link between the commodities prices since de growth of GDP is substantially dependent on the exports that impact the NER. The heatmaps show similar behavior for the NER and NIR correlation. Each time series has significant effects when analyzed with sales, and it can be aimed at examining and validating the results for forecasting sales. The method has also uncovered multifractal regimes with three different exponents (scaling factors) for analyzing pair of macroeconomic time series. In this case, the regimes defined from Region II occur in approximately 15 months, and Region III in nearly 67 months. The exponents derived from the global analysis show positive persistence for NER’s DFA in Region I, GDP for Region II, and NER and GDP for Region III. These persistences show that seasonal regimes can directly impact Brazilian automotive sales. Additionally, the DCCA scaling factor grows from Region I (anti-persistent) to Region III (persistent), except for the DCCA exponent from NER and NIR, which decreases from Region I (persistent) to Region III (anti-persistent). We have also computed the average intensity of $\sigma$ as a function of time. The average $\sigma$ has shown cyclical patterns of 3 years and peaks of positive persistence around 2007, 2010, and 2013. Furthermore, we have found an overall anti-persistence pattern compatible with the argument that unfavorable fluctuations in the NER tend to decrease the prices and possibly increase sales of automotive vehicles in Brazil. Therefore, we have confirmed the reliability of the MF-DCCHM to study cyclic patterns of time series fluctuations across multiple regimes.

## Methods

### Detrended fluctuation analysis

We have employed the detrended fluctuation analysis (DFA)^86,93^ to obtain long-range correlations between time series. The signals are mapped to integrated time series $y_k = \sum _{i=1}^{k} x_i$ and increments $x_i=y_{k+1} -y_k$ with $i,k ~\epsilon ~\{1,2,...,N\}$, where N represents the total number of records. The procedure is to split the series into $M_v$ sliding boxes of size *v* represented by the pair (*m*,*v*) where $1 \le m \le M_v$. The time-series fluctuations are computed from the averages and linear trends over boxes *m* of size *v*, as shown in Fig. S1a,b (Supporting Information). Additionally, the covariance *f*(*m*, *v*) is computed by subtracting $y_k$ from the average for each box *m* according to the following equations:

2 $$\begin{aligned} f^{2}_{DFA_0} (m,v)= & {} \frac{1}{v} \sum _{k=I_{min}(m,v)}^{I_{max}(m,v)} \left[ y_k - \overline{y}_k (m,v)\right] ^2, \end{aligned}$$

3 $$\begin{aligned} f^{2}_{DFA_1} (m,v)= & {} \frac{1}{v} \sum _{k=I_{min}(m,v)}^{I_{max}(m,v)} \left[ y_k - {p}_k (m,v)\right] ^2. \end{aligned}$$

Finally, we computed the average over the fluctuations $F_X^2$ considering all m-*ith* sliding boxes of size *v*, where:

4 $$\begin{aligned} F_X^2 = \frac{1}{M_v} \sum _{m=1}^{M_v} f_X^2(m,v), \end{aligned}$$

where X represent the methods $X=DFA_0$ and $X=DFA_1$. These equations are computed recurrently with sliding boxes of different sizes *v*. We assume the power-law $F_X \approx v^{\alpha }$, where *v* represent the box size and the scaling factor $\alpha$ is obtained by linearization $\log (F_X) X \log (v)$. As a result, we can classify anti-persistent and persistent behavior based on the scaling factor $\alpha$. The tendency can reverse shortly if $0<\alpha <0.5$. However, due to random effects such as white noise, the integrated series has no autocorrelation if the scaling factor $\alpha = 0.5$. The tendency remains persistent for $0.5< \alpha <1.0$, which means the integrated signal continues its prior trend. It is often difficult to determine whether the interference comes from external or internal sources. Therefore, data processing and re-factoring are critical to eliminating possible biases, random trends, and masked signals.

### Detrended cross correlation analysis

The detrended cross-correlation analysis (DCCA)^94^ is a generalization of the DFA technique since it takes the long-range cross-correlation memories of two non-stationary signals with the same size *N*. Consider two signals $y_k$ and ${y'_k}$ with *N* records, and its respective increments $x_i$ and ${x'_i}$, such that:

5 $$\begin{aligned} y_k = \sum _{i=1}^{k} x_i ~~and~~ {y'_k} = \sum _{i=1}^{k} {x'_i}, \end{aligned}$$

where $k = \{1,2,...,N\}$. First, the integrated series splits into $M_v$ sliding boxes of size *v*, where each box is described as (*m*, *v*), where $1 \le m \le M_v$. The fluctuations *f*(*m*, *v*) are computed with the following equations:

6 $$\begin{aligned} f^{2}_{DCCA_0} (m,v)= & {} \frac{1}{v} \sum _{k=I_{min}(m,v)}^{I_{max}(m,v)} \left[ y_k - \overline{y}_k (m,v)\right] \left[ y'_k - \overline{y}'_k (m,v)\right] , \end{aligned}$$

7 $$\begin{aligned} f^{2}_{DCCA_1} (m,v)= & {} \frac{1}{v} \sum _{k=I_{min}(m,v)}^{I_{max}(m,v)} \left[ y_k - {p}_k (m,v)\right] \left[ y'_k - {p}'_k (m,v)\right] , \end{aligned}$$

8 $$\begin{aligned} f^{2}_{|DCCA_1|} (m,v)= & {} \frac{1}{v} \sum _{k=I_{min}(m,v)}^{I_{max}(m,v)} |\left[ y_k - {p}_k (m,v)\right] \left[ y'_k - {p}'_k (m,v)\right] |. \end{aligned}$$

The fluctuations $F_X$ are derived from the Eq. (4), where the sub-index *X* refers to the methods $X=DCCA_0,~DCCA_1$ or $|DCCA_1|$. The procedure is to compute these averages by varying the size of the boxes for the integrated series. In Eq. (2), the parameter $\overline{y}_k(m,v)$ represents the average of $y_k$, where the box (*m*, *v*) is constrained to the interval from $I_{min} (m,v)$ to $I_{max} (m,v)$. Equation (3), $p_k (m,v) = a(m,v) z_k + b(m,v)$ is a first-order polynomial function where the parameters are determined by the method of least squares. This equation represents a linear trend for a specific box represented by the pair of parameters (*m*, *v*). The Eqs. (6)–(8), $\overline{y}_k$ and $\overline{y}'_k$ represent averages over $y_k$ and $y_k'$ by considering a box inside the interval from $I_{min}(m,v)$ to $I_{max}(m,v)$. Furthermore, $p_k(m,v) = a(m,v)z_k + b(m,v)$ and $p_k'(m,v) = a'(m,v)z_k' + b'(m,v)$ is a first degree polynomial, and the notation $|DCCA_1|$ in Eq. (8) represents the absolute value for the local fluctuations of each time series. The fluctuations are represented by the power-law $F_X(v)=v^\lambda$ where the scaling factor $\lambda$ measures the cross-correlations between two signals.

### Spectral analysis

The Discrete Fourier Transform (DFT), considering N terms, is given by:

9 $$\begin{aligned} F_m = \sum _{n=0}^{N} f_n \exp {\left( \frac{-i 2 \pi n \Delta {m}}{N}\right) }, \end{aligned}$$

where $0 \le n \le N$ and $0 \le m \le N$ with increments n and m which are associated with the changes in the interval $\Delta {m}=\frac{1}{N\Delta {n}}$. The DFT is a powerful technique to assess frequency anomalies. We have used the amplitude and power spectrum with the following equation:

10 $$\begin{aligned} A_m =\sqrt{Re F_m^2 + Im F_m^2}, ~~and~~P_m= [A_m]^2. \end{aligned}$$

The power spectrum of a random time series does not show any spectral structure because it is constant. When analyzing the spectra of any time series, our primary focus is to detect and interpret the peaks or anomalies that may exist and their respective frequencies. Additionally, we can use specific cases of the power spectrum to model attributes and obtain a proportionality factor of the power spectrum with the following equation:

11 $$\begin{aligned} P(f) = \frac{1}{f^b}, y(x)=-bx, \end{aligned}$$

where $y=\log [P(f)]$, $x = \log (f)$, and the coefficients are associated with the spectral exponent.

### Multifractal detrended cross-correlation heatmaps

The cross-correlation coefficient (CCC) is a critical factor that quantifies the cross-correlation between two non-stationary signals^87^. The CCC is defined for each window of size *v* with the following ratio:

12 $$\begin{aligned} \sigma _{DCCA_1}(v) = \frac{F^2_{DCCA_1}(v)}{F_{DFA_1}(v) F'_{DFA_1}(v)}, \end{aligned}$$

where $\sigma _{DCCA_1}(v)$ is a dimensionless quantity that varies in the interval $-1 \le \sigma _{DCCA_1} \le 1$. Similar to the standard correlation coefficient, the threshold $\sigma _{DCCA_1} = 1$ represent the maximum cross-correlation, $\sigma _{DCCA_1} = 0$ indicates no-correlation, while $\sigma _{DCCA_1} = -1$ characterizes the maximum anti-cross-correlation. The MF-DCCHM relies on computing the *DCCA* coefficient for multiple windows of size *v* using a sub-series of sliding boxes with size of up to 5% of the entire series, similar to moving average, to observe cross-correlation patterns between the time series for different temporal scales in one single map. This map can also be constructed for a configurational space (spatial/temporal scale). For instance, we can calculate whether a high cross-correlation is valid for all scales or if any intensity change exists for any given scale. The overall sampling of DCCA for multiple time steps and windows of size *v* can help unveil potential cyclical patterns and their consistency. We have also estimated and plotted the average sampling of DCCA over all possible windows of size *v* on top of each cross-correlation heatmap to visualize the trends better.

## Supplementary Information

Supplementary Figures.

## Data Availability

The datasets generated during and/or analysed during the current study are available from the corresponding author on reasonable request.
