# Supplementary material for: Multi-fractal detrended cross-correlation heatmaps for time series analysis
Source: Sci Rep. 2022 Dec 15;12:21655. doi: 10.1038/s41598-022-26207-w (PMC9755263; doi:10.1038/s41598-022-26207-w)
Supplement: Supplementary file 1 — Supplementary Figures. [file 41598_2022_26207_MOESM1_ESM.pdf]

Supporting Information

Multi-Fractal Detrended Cross-Correlation Heatmaps for Time Series Analysis

Paulo Roberto Barros de Melo Junior [1], **Kianny Lopes Bunge** [2], **Vitor Hugo Serravalle Reis Rodrigues** [3], **Michell Thompson Ferreira Santiago** [4], **Euler Bentes dos Santos Marinho** [4], and **Jose Luis Lima de Jesus Silva** [5,+,*]

1. **Petrobras, Petróleo Brasileiro S.A.,** Av. República do Chile, nº 65 Centros, Rio de Janeiro, 20031-912, Brasil
2. **UniFAMEC**, Avenida Leste, Ponto Certo, Camacari-BA, 42801-170, Brazil
3. **Geological Survey of Brasil - CPRM**, Avenida Ulysses Guimar˜aes, 2862 Centro Administrativo da Bahia, Salvador-BA, 1649-026, Brazil
4. **Federal University of Bahia, Polytechnic School of Engineering and Institute of Geosciences, Research Center in Geophysics and Geosciences**, Rua Barão de Jeremoabo, Ondina, Salvador-BA, 40210-630, Brazil
5. **Linköping University,** Department of Computer and Information Science, Division of Artificial Intelligence and Integrated Computer Systems, Linköping, SE-581 83, Sweden

Corresponding author: * jose.silva@liu.se
Data Availability: + The datasets generated during and/or analysed during the current study are available from the corresponding author on reasonable request.

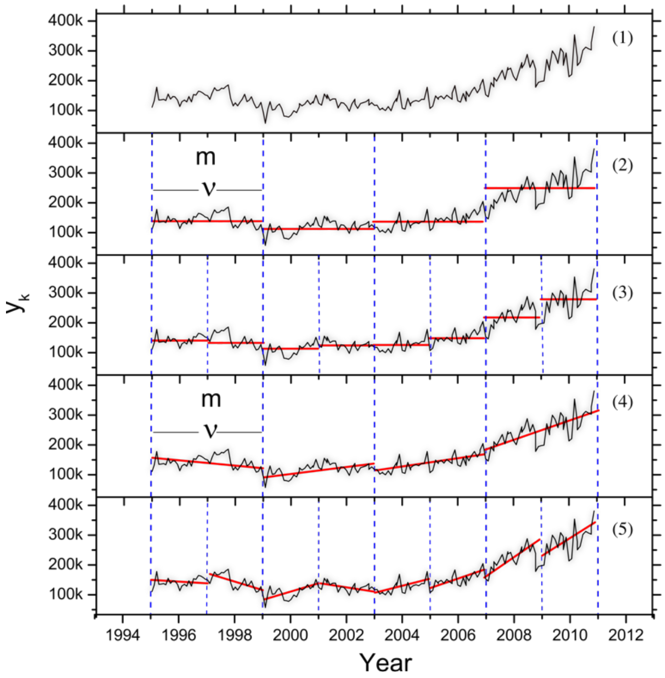

Figure S1: Illustrative steps for Autocorrelation. (1) Time Series of sales y_k in units (Brazilian Automotive Sector), (2) Average (red) of Time series for four windows m of size v, and (3) eight windows. (4) First-order adjustment considering four and (5) eight windows, respectively.

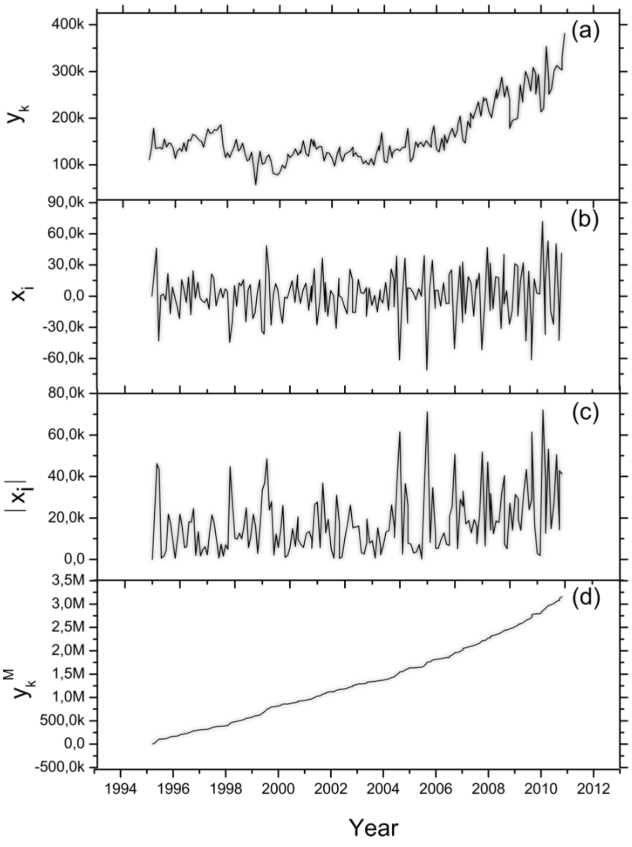

Figure S2: (a) Time series of sales y_k. (b) Time series of successive differences y_k, where x_i = $y_{k+1} - y_k$. (c) Series of magnitudes for successive differences x_i, and (d) Integrated series of magnitudes with y_k = $\sum_{i=1}^{k} |x_i|$.

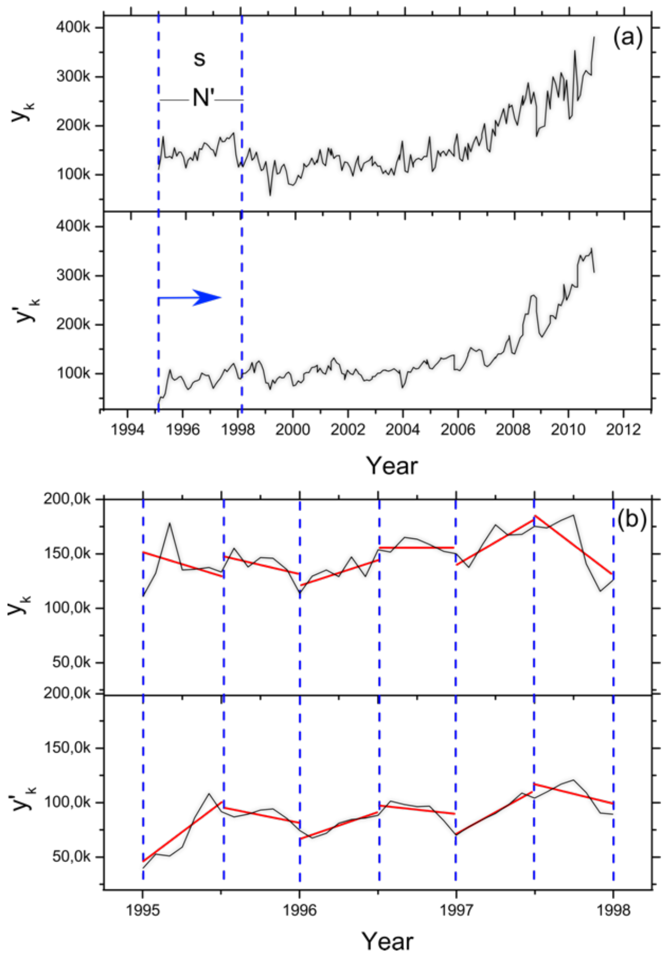

Figure S3: (a) Illustration of the local analysis procedure for sliding boxes of size N. (b) Expansion of the box s divided into six periods m of size v.
